# Supplementary material for: Association between dementia risk and ulcerative colitis, with and without colectomy: a Swedish population-based register study
Source: BMJ Open. 2023 Dec 22;13(12):e074110. doi: 10.1136/bmjopen-2023-074110 (PMC10749019; doi:10.1136/bmjopen-2023-074110)
Supplement: Author's manuscript [file bmjopen-2023-074110.draft_revisions.pdf]

BMJ Open is committed to open peer review. As part of this commitment we make the peer review history of every article we publish publicly available.

When an article is published we post the peer reviewers' comments and the authors' responses online. We also post the versions of the paper that were used during peer review. These are the versions that the peer review comments apply to.

The versions of the paper that follow are the versions that were submitted during the peer review process. They are not the versions of record or the final published versions. They should not be cited or distributed as the published version of this manuscript.

BMJ Open is an open access journal and the full, final, typeset and author-corrected version of record of the manuscript is available on our site with no access controls, subscription charges or pay-per-view fees (http://bmjopen.bmj.com).

If you have any questions on BMJ Open's open peer review process please email info.bmjopen@bmj.com

BMJ Open

Dementia risk and ulcerative colitis, with and without colectomy: a Swedish population-based register study.

Journal:	*BMJ Open*
Manuscript ID	bmjopen-2023-074110
Article Type:	Original research
Date Submitted by the Author:	27-Mar-2023
Complete List of Authors:	Garcia-Argibay, Miguel; Örebro universitet, Clinical Epidemiology and Biostatistics, School of Medical Sciences, Faculty of Medicine and Health Örebro University; Karolinska Institute, Department of Medical Epidemiology and Biostatistics<br>Hiyoshi, Ayako; Orebro Universitet, Clinical Epidemiology and Biostatistics, School of Medical Sciences, Faculty of Medicine and Health; Stockholm University, Department of Public Health Sciences<br>Montgomery, Scott ; Örebro universitet, Clinical Epidemiology and Biostatistics, School of Medical Sciences, Faculty of Medicine and Health; University College London, Department of Epidemiology and Public Health
Keywords:	Inflammatory bowel disease < GASTROENTEROLOGY, Epidemiology < TROPICAL MEDICINE, GASTROENTEROLOGY, Gastroenterology < INTERNAL MEDICINE

SCHOLARONE™
Manuscripts

I, the Submitting Author has the right to grant and does grant on behalf of all authors of the Work (as defined in the below author licence), an exclusive licence and/or a non-exclusive licence for contributions from authors who are: i) UK Crown employees; ii) where BMJ has agreed a CC-BY licence shall apply, and/or iii) in accordance with the terms applicable for US Federal Government officers or employees acting as part of their official duties; on a worldwide, perpetual, irrevocable, royalty-free basis to BMJ Publishing Group Ltd ("BMJ") its licensees and where the relevant Journal is co-owned by BMJ to the co-owners of the Journal, to publish the Work in this journal and any other BMJ products and to exploit all rights, as set out in our licence.

The Submitting Author accepts and understands that any supply made under these terms is made by BMJ to the Submitting Author unless you are acting as an employee on behalf of your employer or a postgraduate student of an affiliated institution which is paying any applicable article publishing charge ("APC") for Open Access articles. Where the Submitting Author wishes to make the Work available on an Open Access basis (and intends to pay the relevant APC), the terms of reuse of such Open Access shall be governed by a Creative Commons licence – details of these licences and which Creative Commons licence will apply to this Work are set out in our licence referred to above.

Other than as permitted in any relevant BMJ Author's Self Archiving Policies, I confirm this Work has not been accepted for publication elsewhere, is not being considered for publication elsewhere and does not duplicate material already published. I confirm all authors consent to publication of this Work and authorise the granting of this licence.

For peer review only

Dementia risk and ulcerative colitis, with and without colectomy: a Swedish population-based register study.

For peer review only

Miguel Garcia-Argibay[1,2*], Ph.D., Ayako Hiyoshi[1,3,4,5], Ph.D., Scott Montgomery[1,4,6], Ph.D.

[1]Clinical Epidemiology and Biostatistics, School of Medical Sciences, Faculty of Medicine and Health, Örebro University, Örebro, Sweden
[2]Department of Medical Epidemiology and Biostatistics, Karolinska Institutet, Stockholm, Sweden
[3]Department of Public Health Sciences, Stockholm University, Stockholm, Sweden
[4]Department of Epidemiology and Public Health, University College London, London, UK
[5]Public Health, Department of Social Medicine, Osaka University Graduate School of Medicine, Osaka, Japan.
[6]Clinical Epidemiology Division, Department of Medicine, Karolinska Institutet, Solna, Sweden

Author notes

*Correspondence concerning this article should be addressed to Miguel Garcia-Argibay, School of Medical Sciences, Örebro University, SE-70182 Örebro, Sweden.
E-mail: Miguel.garcia-argibay@oru.se

Abstract

Objective to investigate the association of ulcerative colitis (UC) with all-cause dementia and assess differences in those with a total colectomy.

Design, setting, and participants This Swedish prospective register-based study comprised 4.8 million individuals aged at least 59 years between 1939 and 2018 with linkage of several Swedish national registers.

Primary and secondary outcome measures: Individuals with dementia were defined according to ICD diagnostic codes and ATC codes for medication prescriptions. Fitting Cox hazards models, the risk of developing all-cause dementia in individuals with and without UC was estimated. Further, we compared the risk of all-cause dementia among those with and without a colectomy.

Results Among 4,821,488 individuals (52.6% females) followed for 84.1 million person-years between 1964 and 2018, the incidence rate of all-cause dementia was 63.90 (63.73–64.07) events per 10,000 person-years in the cohort, 95.01 (92.25–97.86) in those with UC but without colectomy, and 63.42 (40.92–98.31) in those with UC and a colectomy. Adjusted Cox models showed an increased all-cause dementia risk in individuals with UC (HR=1.07 [1.04–1.10]). Compared to unexposed individuals, those with UC and a colectomy showed no increased risk (HR=0.89 [0.57–1.38]).

Conclusion The findings are consistent with previous evidence suggesting a slightly increased dementia risk among individuals with UC. This study provided no evidence of further risk increase of dementia among those who had a colectomy.

Keywords: ulcerative colitis, inflammatory bowel disease, colectomy, epidemiology.

WC: 2249

For peer review only

Strengths and limitations of this study

- This study was based on data from several Swedish registers with over 4.8 million individuals including 86.7 million person-years over seven decades followed for an average of 17 years of observation time.
- Prospectively recorded coverage from inpatient and outpatient services with a 79–90% positive predictive value for UC.
- By using national registers, misclassification of dementia and ulcerative colitis is plausible.
- Due to the limited number of individuals with UC who underwent colectomy, we could not evaluate the significance of age at colectomy and whether this association differs according to the type of dementia.

For peer review only

Introduction

Inflammatory bowel disease (IBD) is a complex, multifactorial disorder with a multifaceted pathogenesis and is believed to result from inappropriate and sustained activation of the mucosal immune system that results in chronic gut inflammation. IBD is a collective term for related intestinal disorders, particularly Crohn's disease (CD) and ulcerative colitis (UC). While CD is characterized by inflammation in any part of the gastrointestinal tract and extraintestinal manifestations, UC is confined to the inner lining of the colon and rectum. Total colectomy is a major bowel surgery used in the treatment of severe and extensive UC that involves the removal of the entire colon and, often, the rectum. This surgical procedure is usually performed among those who do not respond to pharmaceutical treatment or when all the diseased tissues cannot be removed by less extensive surgery such as partial colectomy or hemicolectomy. In addition to providing relief from the disease itself, colectomy can help eliminate the cause of inflammation in the digestive tract.

The association between chronic systemic inflammation and many common psychiatric and physical conditions is well documented.[1–4] High levels of circulating proinflammatory cytokines increase permeability of the blood brain barrier[5,6], thus affecting neurotransmitter systems and altering the metabolism of the central nervous system.[7,8] For instance, inflammatory cytokines (e.g., TNF-α, IL-1, and IL-6) have been associated with several mental conditions including major depressive disorder[9], suicidal ideation[10], vascular dementia[11] and Alzheimer’s disease.[11,12]

It is therefore conceivable that proinflammatory changes in the brain due to UC may mediate some of these effects and be involved in the pathophysiology of dementia. Indeed, prior research indicated an increased risk for developing dementia

For peer review only - http://bmjopen.bmj.com/site/about/guidelines.xhtml

in those with IBD.[13,14] Despite colectomy indicating more aggressive UC behaviour, we hypothesised that colectomy might confer some protection against developing dementia in individuals with UC by eliminating chronic colonic inflammation.

This study used national Swedish register data to 1) assess the risk of developing dementia in individuals with and without UC; 2) compare this risk in those with UC who underwent colectomy with those who did not.

Methods

Study design and population

This study was based on several Swedish national registers linked through the personal identification number issued to all residents in Sweden. The Total Population Register (TPR) includes demographic information such as date of birth, sex, date of death, and migration for all individuals born since 1932 and alive in 1963 and beyond.[15] The National Patient Register (NPR) records primary and secondary diagnoses from inpatient care since 1964 and outpatient information since 2001.[16] The Cause of Death Register (CDR) contains information on all deaths since 1952.[17] In both the NPR and CDR, diagnoses were classified according to the International Classification of Diseases (ICD) versions 7/8/9/10. Additionally, the Prescribed Drug Register (PDR) provided data on all dispensed medication prescriptions since July 1, 2005, including prescription date and dosage, utilizing the Anatomical Therapeutic Classification (ATC) system. Lastly, the Longitudinal integration database for health insurance and labour market studies register (LISA) and the Population and Housing Census were used to obtain socioeconomic information.

For peer review only

We identified all individuals born in Sweden before or in 1958. Individuals were followed from age 59 years or the beginning of the NPR on January 1, 1964 (whichever occurred last), until death, emigration, a diagnosis of dementia, or the end of the study period (December 31, 2018), whichever occurred first. Individuals with less than one year of follow-up were excluded.

Patient and public involvement

No patient involved. The public was also not involved in the design, or conduct, or reporting, or dissemination plans of the research.

Outcome

All-cause dementia was defined as either 1) a recorded diagnoses in the NPR using ICD codes: ICD-7: 304, 305 and 306, ICD-8 290, 293.0-293.1, ICD-9 290A, 290B, 290E, 290W, 290X, 294B, 331A-C, 331X, and ICD-10 F00-F01, F02.3, F03, F05.1, F09, G30, or G31.1, 2) at least two recorded dispensed medications of any of the following anticholinesterases: N06DA01, N06DA03, N06DA04, N06DX01, N06DA02, N06DA52, or 3) a recorded entry in the Death register with the previous all-cause dementia ICD codes. As we only had access to three-digit codes for ICD-8, differentiating between UC and CD relied on subsequent diagnoses in the same patients using ICD-9/10 codes. Those individuals who were solely identified through ICD-8 codes were excluded from the analysis. Given the insidious nature of dementia, the date of the onset of all-cause dementia was defined as three years before the diagnosis of dementia in the NPR or a dispensed medication and five years before the death. Individuals with a diagnosis of dementia before the start of follow-up were excluded from the analysis.

For peer review only

Exposures

In this study, we included two different exposures: the occurrence of UC, with or without colectomy. The date of diagnosis of UC was identified using ICD codes in the NPR: ICD-7: 572, ICD-8 563, 569, ICD-9 556, and ICD-10 K51. Individuals who were initially diagnosed with UC followed by two CD diagnoses were classified as having CD from the first UC diagnosis and censored at that date. Likewise, those who had a CD diagnosis first, but followed by two or more UC diagnoses were classified as having UC from the first diagnosis. Among individuals with UC, we identified individuals who underwent total colectomy and were identified using the Swedish Classification of Operation and Major Procedures codes: JFH00, JFH01, JFH10, JFH11, JFH20, JFH30, JFH33, JFH40, and JFH96. Both exposures were allowed to vary over time, that is, individuals started as nonexposed until the occurrence of each exposure separately and considered as exposed from that time onwards. Those who had UC or a colectomy before the start of follow-up were coded as exposed from the start of follow-up. We created two variables: one indicating the presence or absence of a UC diagnosis (1/0) and a second variable further breaking down people with UC into with and without colectomy and compared both groups to those without UC.

Covariates

Potential confounding factors included birth year, sex, region (Norrland, Svealand, and Götaland), socioeconomic index (SEI), and the household crowding index (HCI). Using the Population and Housing Census (FOB; 1960–1990 every 10 years), we extracted information on SEI and HCI using the year closest to birth (when not available, information was taken from the census up to age 10 years). SEI was based on the occupation of the father of the household (or the mother when there

For peer review only

was no information on the father). The HCI was calculated as the ratio of total number of individuals in the household to the number of rooms in the household.

Statistical analysis

Demographic and other individual baseline characteristics were summarized by median, proportions, and interquartile range (IQR) for the overall cohort and for individuals with UC that did and did not undergo colectomy. Incidence rates (IR) of dementia with 95% confidence intervals (CI) were calculated fitting a Poisson generalized linear model with the log link function and robust standard errors. A Cox proportional hazards model with age as the underlying time scale[18] was fitted to estimate hazard ratios (HRs) with 95% confidence intervals (CI) for the association between colectomy and the development of dementia. Both exposures (UC and total colectomy) were modelled as time-dependent covariates, that is, individuals were classified as exposed only after the occurrence of each exposure. Analyses were adjusted for sex, birth year, and county of birth. Shoenfeld residuals were inspected in order to assess the proportionality assumption and when violated, analyses were stratified by the variables that did not meet this assumption. Data management and statistical analyses were performed using R 4.2.2.[19]

Sensitivity analysis

Due to incomplete coverage in the NPR, the analysis was repeated including only individuals who were 60 years old after 1973 to ensure greater coverage.

Results

This cohort study comprised 4,821,488 individuals with a median (IQR) age at the start of follow-up of 59 (SD=0.71) years, of whom 2,534,500 (52.6%) were

For peer review only

female and 2,286,988 (47.4%) were male. Among those, 48,993 (1%) had a diagnosis of UC, of whom 493 (1%) had a colectomy before or during follow-up, with a median (IQR) age at the operation of 63 (51–74) years. During a total follow-up time of 84.1 million person-years between 1939 and 2018, a diagnosis of all-cause dementia was observed in 546,387 (11.3%) individuals. A summary of the cohort demographic characteristics is presented in Table 1.

The incidence rate of all-cause dementia was 63.90 (63.73–64.07) events per 10,000 person-years in the cohort, 95.01 (92.25–97.86) in those with UC but without colectomy, and 63.42 (40.92–98.31) for those without UC. Cox models adjusted for sex, birth year, and birth county showed that individuals with UC had an increased all-cause dementia risk compared with the general population (HR=1.07, 95% CI [1.04–1.10]). When we separated individuals with UC into with or without a colectomy and compared both groups to individuals without UC, dementia risk for individuals with UC but without colectomy is increased (HR=1.07 [1.04–1.10]), but the risk became nonsignificant for individuals with UC and a total colectomy (HR=0.89 [0.57–1.38]). Sensitivity analyses showed that after restricting analyses to those who started follow-up in 1974 and onwards, the magnitude of the association decreased compared with the main analysis but still indicated that those with UC were at increased risk for all-cause dementia (HR=1.04 [1.01–1.08]). For those with UC and without a colectomy, we observed an increased risk for all-cause dementia compared to the general cohort (HR=1.04 [1.01–1.08]) and no difference in the hazard between those with UC and a total colectomy and the general population (HR=0.92 [0.60–1.43]). Figure 1 summarizes the results of the main and sensitivity analyses.

For peer review only

Discussion

This large cohort study of individuals from the general population with prospectively recorded data assessed the association between UC and subsequent development of all-cause dementia and investigated the previous unexplored topic, to the best of our knowledge of the potential role of colectomy in this relationship. The results indicated that individuals with UC are at slightly increased all-cause dementia risk compared with those without UC, but there was no evidence of an even greater magnitude risk in those who has a colectomy.

Recent meta-analysis evidence indicated an increased dementia risk in individuals with IBD with a pooled risk ratio of 1.35 [1.08–1.68].[14] Our findings are consistent with this finding and mirror conclusions obtained in previous studies suggesting a relationship between UC and dementia, with a similar magnitude of the association to those obtained in Danish register studies[13] and Canadian[20] and German cohort studies[21]. Our study provides novel findings by indicating that having a total colectomy does not further increase dementia among people with UC. While our results are consistent with possible reduction of dementia risk in UC following colectomy, the interpretation remains equivocal. It is noteworthy that as colectomy signals more severe UC disease activity, as well as the inconvenience post-colectomy, there was no additional dementia risk compared to those with UC without colectomy.

While the precise aetiology of UC remains elusive, it has been proposed that a dysregulated mucosal immune response, in the context of a disturbed gut microbiota, plays a pivotal role in the onset and progression of the disease.[22–25] The intestinal immune response is a process composed of complex interactions between several cell

For peer review only

types interacting within a specific microenvironment, the intestinal mucosa. When these interactions are perturbed by dysbiosis, such as during the onset of IBD, an excessive immune response occurs, with an aberrant mucosal immune response that involves the production of large amounts of pro-inflammatory cytokines, such as interleukins, that can lead to severe tissue damage[26] and it has been associated with several psychiatric conditions, including dementia.[1–4,9–12] A balance between proinflammatory and anti-inflammatory signals is necessary for the host to avoid acute or chronic inflammation and its harmful consequences. We hypothesize that with the removal of the colon, the excessive immune response and the overproduction of pro-inflammatory cytokines cease, reducing colonic and potentially chronic systemic inflammation and, in turn, potentially reducing the risk of dementia.

Strengths and limitations

This study presents several noteworthy strengths, including a nationwide sample of over 4.8 million individuals including 86.7 million person-years over seven decades followed for an average of 17 years of observation time with prospectively recorded coverage from inpatient and outpatient services with a 79–90% positive predictive value for UC.[27] To further reduce UC misclassification, we required patients to have at least 2 consecutive UC diagnoses. There are potential limitations that should be considered. First, the estimated increased risk for dementia in people with UC may be, in part, explained by surveillance bias, where people with UC have increased contact with healthcare systems. However, those with UC and a colectomy also experience closer contact with healthcare services and we did not observe an increased risk. Second, although we obtained all-cause dementia diagnoses from inpatient and outpatient care services, misclassification is possible. In order to reduce

For peer review only

misclassification, we retrieved all prescribed anticholinesterases used to treat dementia from the PDR from 2005 onwards (>99% coverage). Nonetheless, our estimated IR (6.4 per 1,000 person-year) is smaller than those recently reported in the literature[28], highlighting potential misclassification. This misclassification –most likely non-differential– refers to individuals with dementia that are not identified leading to systematic error in the estimation of association and would bias results towards the null. Further, given the low count of individuals with UC who had a colectomy, we were not able to assess the importance of age at colectomy and whether this association varies by dementia type.

Conclusions

Our findings contribute to the growing body of literature on the increased risk of developing dementia among individuals with UC. Additionally, our study indicated that total colectomy in UC patients is not associated with an even higher magnitude increased dementia risk.

For peer review only

Author Contributions

Dr Garcia-Argibay had full access to all the data in the study and takes responsibility for the integrity of the data and the accuracy of the data analysis.

Concept and design: All authors.

Statistical analysis: Garcia-Argibay.

Acquisition, analysis, or interpretation of data: All authors.

Drafting of the manuscript: Garcia-Argibay.

Critical revision of the manuscript for important intellectual content: All authors.

Supervision: Montgomery, Hiyoshi.

Conflict of Interest Disclosures

Funding/Support

This work was supported by grant from the Swedish Research Council for Health, Working Life and Welfare (number: 2019-01236). AH's participation was also supported by Osaka University International Joint Research Promotion Program (Type A) 2019-2022 with University College London: Integrated research on prevention, treatment and care for dementia.

Role of the Funder/Sponsor

The funders had no role in the design and conduct of the study; collection, management, analysis, and interpretation of the data; preparation, review, or approval of the manuscript; and decision to submit the manuscript for publication.

For peer review only

Ethical approval

The study had ethical approval from the Regional Ethical Review Board in Stockholm, Sweden (Dnr 2019-04755, 2020-02406, and 2022-00336-02). Requirement for informed consent was waived for the current study because it was a secondary analysis of existing data. The investigation conforms to the 1964 Helsinki declaration and its later amendments or comparable ethical standards.

Data sharing

The Public Access to Information and Secrecy Act in Sweden prohibits us from making individual level data publicly available. Researchers who are interested in replicating our work can apply for individual level data at Statistics Sweden: www.scb.se/en/services/guidance-for-researchers-and-universities/.

For peer review only

References

1 Netea MG, Balkwill F, Chonchol M, *et al.* A guiding map for inflammation. *Nat Immunol* 2017;**18**:826–31. doi:10.1038/ni.3790

2 Bennett JM, Reeves G, Billman GE, *et al.* Inflammation–Nature's Way to Efficiently Respond to All Types of Challenges: Implications for Understanding and Managing "the Epidemic" of Chronic Diseases. *Front Med* 2018;**5**:316. doi:10.3389/fmed.2018.00316

3 Walker KA, Gottesman RF, Wu A, *et al.* Systemic inflammation during midlife and cognitive change over 20 years: The ARIC Study. *Neurology* 2019;**92**:e1256–67. doi:10.1212/WNL.0000000000007094

4 Cunningham C, Hennessy E. Co-morbidity and systemic inflammation as drivers of cognitive decline: new experimental models adopting a broader paradigm in dementia research. *Alzheimers Res Ther* 2015;**7**:33. doi:10.1186/s13195-015-0117-2

5 Pan W, P. Stone K, Hsuchou H, *et al.* Cytokine Signaling Modulates Blood-Brain Barrier Function. *Curr Pharm Des* 2011;**17**:3729–40. doi:10.2174/138161211798220918

6 Varatharaj A, Galea I. The blood-brain barrier in systemic inflammation. *Brain Behav Immun* 2017;**60**:1–12. doi:10.1016/j.bbi.2016.03.010

7 Miller AH, Haroon E, Raison CL, *et al.* CYTOKINE TARGETS IN THE BRAIN: IMPACT ON NEUROTRANSMITTERS AND NEUROCIRCUITS: Review: Cytokine Targets in the Brain. *Depress Anxiety* 2013;**30**:297–306. doi:10.1002/da.22084

8 Raison CL, Capuron L, Miller AH. Cytokines sing the blues: inflammation and the pathogenesis of depression. *Trends Immunol* 2006;**27**:24–31. doi:10.1016/j.it.2005.11.006

9 Felger JC, Lotrich FE. Inflammatory cytokines in depression: Neurobiological mechanisms and therapeutic implications. *Neuroscience* 2013;**246**:199–229. doi:10.1016/j.neuroscience.2013.04.060

10 Miná VAL, Lacerda-Pinheiro SF, Maia LC, *et al.* The influence of inflammatory cytokines in physiopathology of suicidal behavior. *J Affect Disord* 2015;**172**:219–30. doi:10.1016/j.jad.2014.09.057

11 Tarkowski E, Blennow K, Wallin A, *et al.* Intracerebral production of tumor necrosis factor-alpha, a local neuroprotective agent, in Alzheimer disease and vascular dementia. *J Clin Immunol* 1999;**19**:223–30. doi:10.1023/a:1020568013953

12 Grimaldi LM, Casadei VM, Ferri C, *et al.* Association of early-onset Alzheimer's disease with an interleukin-1alpha gene polymorphism. *Ann Neurol* 2000;**47**:361–5.

For peer review only

13 Rønnow Sand J, Troelsen FS, Horváth-Puhó E, *et al.* Risk of dementia in patients with inflammatory bowel disease: a Danish population-based study. *Aliment Pharmacol Ther* 2022;**56**:831–43. doi:10.1111/apt.17119

14 Zhang M, Shi Y, Jiang H. The risk of dementia in patients with inflammatory bowel disease: a systematic review and meta-analysis. *Int J Colorectal Dis* 2022;**37**:769–75. doi:10.1007/s00384-022-04131-9

15 Ludvigsson JF, Almqvist C, Bonamy A-KE, *et al.* Registers of the Swedish total population and their use in medical research. *Eur J Epidemiol* 2016;**31**:125–36. doi:10.1007/s10654-016-0117-y

16 Ludvigsson JF, Andersson E, Ekbom A, *et al.* External review and validation of the Swedish national inpatient register. *BMC Public Health* 2011;**11**:450. doi:10.1186/1471-2458-11-450

17 Brooke HL, Talbäck M, Hörnblad J, *et al.* The Swedish cause of death register. *Eur J Epidemiol* 2017;**32**:765–73. doi:10.1007/s10654-017-0316-1

18 Kom EL, Graubard BI, Midthune D. Time-to-Event Analysis of Longitudinal Follow-up of a Survey: Choice of the Time-scale. *Am J Epidemiol* 1997;**145**:72–80. doi:10.1093/oxfordjournals.aje.a009034

19 R Core Team. R: A Language and Environment for Statistical Computing. 2020.

20 Bernstein CN, Nugent Z, Shaffer S, *et al.* Comorbidity before and after a diagnosis of inflammatory bowel disease. *Aliment Pharmacol Ther* 2021;**54**:637–51. doi:10.1111/apt.16444

21 Zingel R, Bohlken J, Kostev K. Association Between Inflammatory Bowel Disease and Dementia: A Retrospective Cohort Study. *J Alzheimers Dis JAD* 2021;**80**:1471–8. doi:10.3233/JAD-210103

22 Targan SR, Karp LC. Defects in mucosal immunity leading to ulcerative colitis. *Immunol Rev* 2005;**206**:296–305. doi:10.1111/j.0105-2896.2005.00286.x

23 Strober W, Fuss I, Mannon P. The fundamental basis of inflammatory bowel disease. *J Clin Invest* 2007;**117**:514–21. doi:10.1172/JCI30587

24 Garrett WS, Lord GM, Punit S, *et al.* Communicable Ulcerative Colitis Induced by T-bet Deficiency in the Innate Immune System. *Cell* 2007;**131**:33–45. doi:10.1016/j.cell.2007.08.017

25 Chen H, Li H, Liu Z. Interplay of intestinal microbiota and mucosal immunity in inflammatory bowel disease: a relationship of frenemies. *Ther Adv Gastroenterol* 2020;**13**:175628482093518. doi:10.1177/1756284820935188

26 Muthas D, Reznichenko A, Balendran CA, *et al.* Neutrophils in ulcerative colitis: a review of selected biomarkers and their potential therapeutic implications. *Scand J Gastroenterol* 2017;**52**:125–35. doi:10.1080/00365521.2016.1235224

For peer review only

27 Jakobsson GL, Sternegård E, Olén O, *et al.* Validating inflammatory bowel disease (IBD) in the Swedish National Patient Register and the Swedish Quality Register for IBD (SWIBREG). *Scand J Gastroenterol* 2017;**52**:216–21. doi:10.1080/00365521.2016.1246605

28 Wolters FJ, Chibnik LB, Waziry R, *et al.* Twenty-seven-year time trends in dementia incidence in Europe and the United States: The Alzheimer Cohorts Consortium. *Neurology* 2020;**95**:e519–31. doi:10.1212/WNL.0000000000010022

For peer review only

Tables

Table 1. Characteristics of the cohort (N=4,821,488).

Variable	Without IBD (N=4,772,495)[1]	UC without colectomy[2] (N=48,500)[1]	UC with colectomy[2] (N=493)[1]
Median follow-up time (years)	17 (10, 25)	17 (10, 24)	13 (7, 19)
Age at the start of follow-up (years)	59 (59, 59)	59 (59, 59)	59 (59, 59)
Sex (%)			
Male	2,263,178 (47)	23,513 (48)	297 (60)
Female	2,509,317 (53)	24,987 (52)	196 (40)
Household crowding index (HCI)	1.50 (1.00, 2.00)	1.50 (1.00, 2.00)	1.50 (1.20, 2.00)
Missing	2,917,369	27,917	186
Socioeconomic index (SEI; %)			
Agriculture	289,162 (6.1)	2,991 (6.2)	43 (8.7)
High	212,356 (4.4)	2,315 (4.8)	35 (7.1)
Low	801,437 (17)	9,268 (19)	138 (28)
Medium	463,642 (9.7)	4,972 (10)	73 (15)
Unknown	2,918,081 (61)	27,919 (58)	186 (38)
Other	87,817 (1.8)	1,035 (2.1)	18 (3.7)
County (%)			
Goaland	2,126,607 (45)	22,223 (46)	190 (39)
Norrland	679,216 (14)	7,004 (14)	52 (11)
Svealand	1,657,347 (35)	17,397 (36)	232 (47)
Unknown	309,325 (6.5)	1,876 (3.9)	19 (3.9)
Median age of colectomy (years)	73 (67, 79)	0	65 (56, 72)
Without colectomy	4,771,206	48,500	0
Dementia (%)	541,574 (11)	4,785 (9.9)	28 (5.7)

Note. [1]Median (IQR); n (%), [2] variables defined as present from the start of follow-up.

For peer review only

Figures

Figure 1. Forest plot of the association between UC and all-cause dementia in those with UC (with and without total colectomy) compared with unexposed individuals.

For peer review only

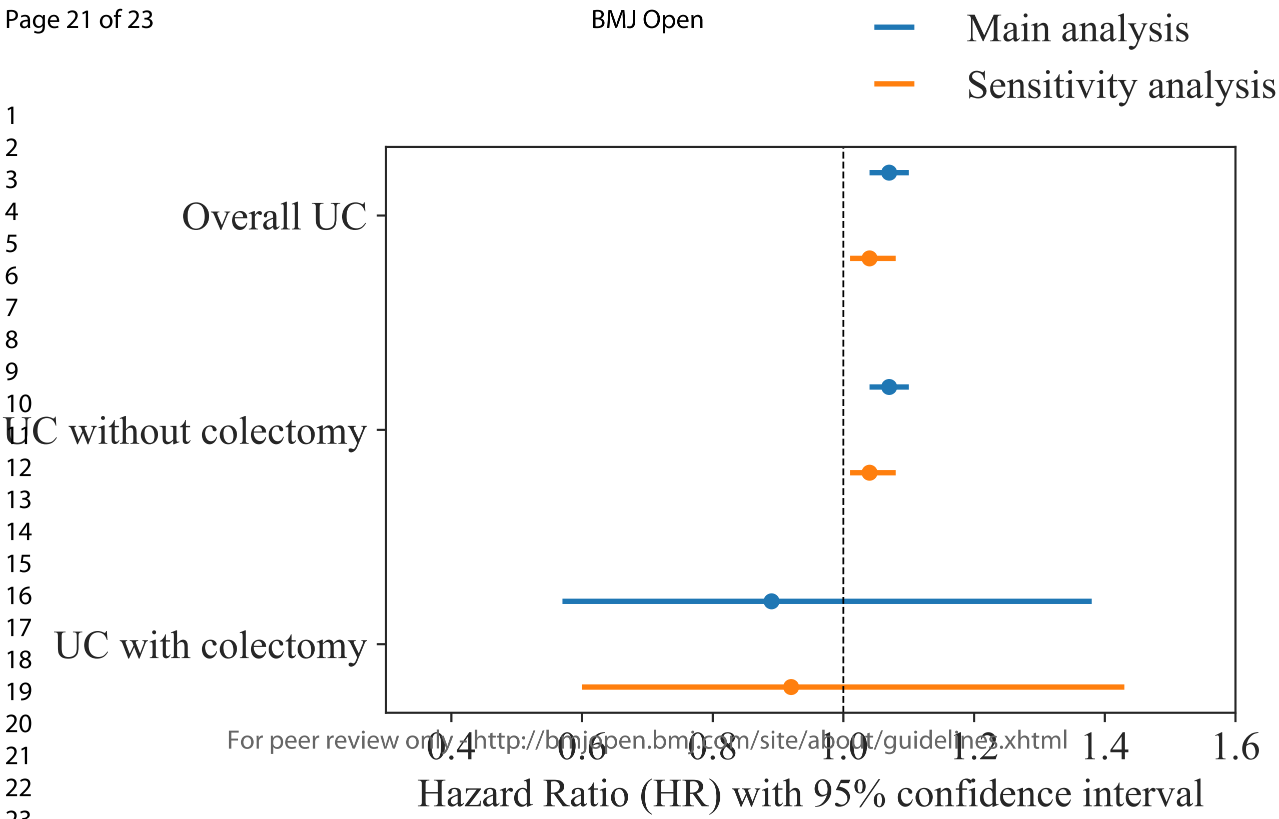
Main analysis
Sensitivity analysis
Overall UC
UC without colectomy
UC with colectomy
0.4
0.6
0.8
1.0
1.2
1.4
1.6
Hazard Ratio (HR) with 95% confidence interval

STROBE Statement—checklist of items that should be included in reports of observational studies

	Item No.	Recommendation	Page No.	Relevant text from manuscript
**Title and abstract**	1	(*a*) Indicate the study's design with a commonly used term in the title or the abstract	1	
		(*b*) Provide in the abstract an informative and balanced summary of what was done and what was found	2	
**Introduction**				
Background/rationale	2	Explain the scientific background and rationale for the investigation being reported	3-4	introduction
Objectives	3	State specific objectives, including any prespecified hypotheses	4	
**Methods**				
Study design	4	Present key elements of study design early in the paper	4	Study design and population
Setting	5	Describe the setting, locations, and relevant dates, including periods of recruitment, exposure, follow-up, and data collection	5	
Participants	6	(*a*) *Cohort study*—Give the eligibility criteria, and the sources and methods of selection of participants. Describe methods of follow-up<br>*Case-control study*—Give the eligibility criteria, and the sources and methods of case ascertainment and control selection. Give the rationale for the choice of cases and controls<br>*Cross-sectional study*—Give the eligibility criteria, and the sources and methods of selection of participants	4-5	
		(*b*) *Cohort study*—For matched studies, give matching criteria and number of exposed and unexposed<br>*Case-control study*—For matched studies, give matching criteria and the number of controls per case		
Variables	7	Clearly define all outcomes, exposures, predictors, potential confounders, and effect modifiers. Give diagnostic criteria, if applicable	4-6	Outcome, exposures, covariates
Data sources/ measurement	8*	For each variable of interest, give sources of data and details of methods of assessment (measurement). Describe comparability of assessment methods if there is more than one group	*4-6*	
Bias	9	Describe any efforts to address potential sources of bias	7	
Study size	10	Explain how the study size was arrived at	4-6	

Continued on next page

For peer review only - http://bmjopen.bmj.com/site/about/guidelines.xhtml

Quantitative variables	11	Explain how quantitative variables were handled in the analyses. If applicable, describe which groupings were chosen and why	4-6	
Statistical methods	12	(*a*) Describe all statistical methods, including those used to control for confounding	7	Statistical analysis
		(*b*) Describe any methods used to examine subgroups and interactions	7	
		(*c*) Explain how missing data were addressed	NA	
		(*d*) *Cohort study*—If applicable, explain how loss to follow-up was addressed<br>*Case-control study*—If applicable, explain how matching of cases and controls was addressed<br>*Cross-sectional study*—If applicable, describe analytical methods taking account of sampling strategy	7	
		(*e*) Describe any sensitivity analyses		
**Results**				
Participants	13*	(a) Report numbers of individuals at each stage of study—eg numbers potentially eligible, examined for eligibility, confirmed eligible, included in the study, completing follow-up, and analysed	7-8	Results
		(b) Give reasons for non-participation at each stage	7-8	
		(c) Consider use of a flow diagram	NA	
Descriptive data	14*	(a) Give characteristics of study participants (eg demographic, clinical, social) and information on exposures and potential confounders	7-8/Table1	
		(b) Indicate number of participants with missing data for each variable of interest	Table1	
		(c) *Cohort study*—Summarise follow-up time (eg, average and total amount)	7-8	
Outcome data	15*	*Cohort study*—Report numbers of outcome events or summary measures over time		
		Case-control study—Report numbers in each exposure category, or summary measures of exposure		
		Cross-sectional study—Report numbers of outcome events or summary measures		
Main results	16	(*a*) Give unadjusted estimates and, if applicable, confounder-adjusted estimates and their precision (eg, 95% confidence interval). Make clear which confounders were adjusted for and why they were included	7-8	
		(*b*) Report category boundaries when continuous variables were categorized		
		(*c*) If relevant, consider translating estimates of relative risk into absolute risk for a meaningful time period		

Continued on next page

For peer review only - http://bmjopen.bmj.com/site/about/guidelines.xhtml

Other analyses	17	Report other analyses done—eg analyses of subgroups and interactions, and sensitivity analyses	8	
**Discussion**				
Key results	18	Summarise key results with reference to study objectives	9-11	Discussion
Limitations	19	Discuss limitations of the study, taking into account sources of potential bias or imprecision. Discuss both direction and magnitude of any potential bias	10	
Interpretation	20	Give a cautious overall interpretation of results considering objectives, limitations, multiplicity of analyses, results from similar studies, and other relevant evidence	10	
Generalisability	21	Discuss the generalisability (external validity) of the study results	10	
**Other information**				
Funding	22	Give the source of funding and the role of the funders for the present study and, if applicable, for the original study on which the present article is based	12	

*Give information separately for cases and controls in case-control studies and, if applicable, for exposed and unexposed groups in cohort and cross-sectional studies.

Note: An Explanation and Elaboration article discusses each checklist item and gives methodological background and published examples of transparent reporting. The STROBE checklist is best used in conjunction with this article (freely available on the Web sites of PLoS Medicine at http://www.plosmedicine.org/, Annals of Internal Medicine at http://www.annals.org/, and Epidemiology at http://www.epidem.com/). Information on the STROBE Initiative is available at www.strobe-statement.org.

For peer review only

BMJ Open

Association between dementia risk and ulcerative colitis, with and without colectomy: a Swedish population-based register study

Journal:	*BMJ Open*
Manuscript ID	bmjopen-2023-074110.R1
Article Type:	Original research
Date Submitted by the Author:	20-Jun-2023
Complete List of Authors:	Garcia-Argibay, Miguel; Örebro universitet, Clinical Epidemiology and Biostatistics, School of Medical Sciences, Faculty of Medicine and Health Örebro University; Karolinska Institute, Department of Medical Epidemiology and Biostatistics<br>Hiyoshi, Ayako; Orebro Universitet, Clinical Epidemiology and Biostatistics, School of Medical Sciences, Faculty of Medicine and Health; Stockholm University, Department of Public Health Sciences<br>Montgomery, Scott ; Örebro universitet, Clinical Epidemiology and Biostatistics, School of Medical Sciences, Faculty of Medicine and Health; University College London, Department of Epidemiology and Public Health
**Primary Subject Heading**:	Epidemiology
Secondary Subject Heading:	Oncology
Keywords:	Inflammatory bowel disease < GASTROENTEROLOGY, Epidemiology < TROPICAL MEDICINE, GASTROENTEROLOGY, Gastroenterology < INTERNAL MEDICINE

SCHOLARONE™
Manuscripts

I, the Submitting Author has the right to grant and does grant on behalf of all authors of the Work (as defined in the below author licence), an exclusive licence and/or a non-exclusive licence for contributions from authors who are: i) UK Crown employees; ii) where BMJ has agreed a CC-BY licence shall apply, and/or iii) in accordance with the terms applicable for US Federal Government officers or employees acting as part of their official duties; on a worldwide, perpetual, irrevocable, royalty-free basis to BMJ Publishing Group Ltd ("BMJ") its licensees and where the relevant Journal is co-owned by BMJ to the co-owners of the Journal, to publish the Work in this journal and any other BMJ products and to exploit all rights, as set out in our licence.

The Submitting Author accepts and understands that any supply made under these terms is made by BMJ to the Submitting Author unless you are acting as an employee on behalf of your employer or a postgraduate student of an affiliated institution which is paying any applicable article publishing charge ("APC") for Open Access articles. Where the Submitting Author wishes to make the Work available on an Open Access basis (and intends to pay the relevant APC), the terms of reuse of such Open Access shall be governed by a Creative Commons licence – details of these licences and which Creative Commons licence will apply to this Work are set out in our licence referred to above.

Other than as permitted in any relevant BMJ Author's Self Archiving Policies, I confirm this Work has not been accepted for publication elsewhere, is not being considered for publication elsewhere and does not duplicate material already published. I confirm all authors consent to publication of this Work and authorise the granting of this licence.

For peer review only

Association between dementia risk and ulcerative colitis, with and without colectomy: a Swedish population-based register study.

For peer review only

Miguel Garcia-Argibay[1,2*], Ph.D., Ayako Hiyoshi[1,3], Ph.D., Scott Montgomery[1,4,5], Ph.D.

[1]Clinical Epidemiology and Biostatistics, School of Medical Sciences, Faculty of Medicine and Health, Örebro University, Örebro, Sweden
[2]Department of Medical Epidemiology and Biostatistics, Karolinska Institutet, Stockholm, Sweden
[3]Department of Public Health Sciences, Stockholm University, Stockholm, Sweden
[4]Department of Epidemiology and Public Health, University College London, London, UK
[5]Clinical Epidemiology Division, Department of Medicine, Karolinska Institutet, Solna, Sweden

Author notes

*Correspondence concerning this article should be addressed to Miguel Garcia-Argibay, School of Medical Sciences, Örebro University, SE-70182 Örebro, Sweden. E-mail: Miguel.garcia-argibay@oru.se

Abstract

Objective To investigate the association of ulcerative colitis (UC) with all-cause dementia and assess differences in those with and without a total colectomy.

Design, setting, and participants This Swedish prospective register-based study comprised 4.8 million individuals aged at least 59 years between 1964 and 2018 with linkage of several Swedish national registers.

Primary and secondary outcome measures Individuals with dementia were defined according to ICD diagnostic codes and Anatomical Therapeutic Classification (ATC) codes for medication prescriptions. Fitting Cox hazards models, the risk of developing all-cause dementia in individuals with and without UC was estimated. Further, we compared the risk of all-cause dementia among those with and without a colectomy.

Results Among 4,821,488 individuals (52.6% females) followed for 84.1 million person-years between 1964 and 2018, the incidence rate of all-cause dementia was 63.90 (63.73-64.07) events per 10,000 person-years in individuals without UC, 94.80 (92.04-97.64) among those with UC, 95.01 (92.25-97.86) in those with UC but without colectomy, and 63.42 (40.92-98.31) in those with UC and a colectomy. Adjusted Cox models showed an increased all-cause dementia risk in individuals with UC (Hazard ratio [HR]=1.07 [1.04-1.10]). We found no differences between unexposed individuals and those with UC and a colectomy (HR=0.89 [0.57-1.38]).

Conclusion The findings are consistent with previous evidence suggesting a slightly increased dementia risk among individuals with UC. This study provided no evidence of further risk increase of dementia among those who had a colectomy.

Keywords ulcerative colitis, inflammatory bowel disease, colectomy, epidemiology.

Word count: 2608

For peer review only

Strengths and limitations of this study

- This study was based on data from several Swedish registers with over 4.8 million individuals including 84.1 million person-years over seven decades followed for an average of 17 years of observation time.
- Prospectively recorded coverage from inpatient and outpatient services with a 79–90% positive predictive value for ulcerative colitis (UC).
- By using national registers, misclassification of dementia and ulcerative colitis is plausible.
- Due to the limited number of individuals with UC who underwent colectomy, we could not evaluate the significance of age at colectomy and whether this association differs according to the type of dementia.

For peer review only

Introduction

Inflammatory bowel disease (IBD) is a complex, multifactorial disorder with a multifaceted pathogenesis and is believed to result from inappropriate and sustained activation of the mucosal immune system that results in chronic gut inflammation. IBD is a collective term for related intestinal disorders, particularly Crohn's disease (CD) and ulcerative colitis (UC). While CD is characterized by inflammation in any part of the gastrointestinal tract and extraintestinal manifestations, UC is confined to the inner lining of the colon and rectum. Total colectomy is a major bowel surgery used in the treatment of severe and extensive UC that involves the removal of the entire colon and, often, the rectum. This surgical procedure is usually performed among those who do not respond to pharmaceutical treatment or when all the diseased tissues cannot be removed by less extensive surgery such as partial colectomy or hemicolectomy. In addition to providing relief from the disease itself, colectomy can help eliminate the cause of inflammation in the digestive tract.

The association between chronic systemic inflammation and many common psychiatric and physical conditions is well documented [1–4]. High levels of circulating proinflammatory cytokines increase permeability of the blood brain barrier [5,6], thus affecting neurotransmitter systems and altering the metabolism of the central nervous system [7,8]. For instance, inflammatory cytokines (e.g., TNF-α, IL-1, and IL-6) have been associated with several mental conditions including major depressive disorder [9], suicidal ideation [10], vascular dementia [11] and Alzheimer's disease [11,12].

It is therefore conceivable that proinflammatory changes in the brain due to UC may mediate some of these effects and be involved in the pathophysiology of

For peer review only

dementia. Indeed, prior research indicated an increased risk for developing dementia in those with IBD [13,14]. Despite colectomy indicating more aggressive UC behaviour, we hypothesised that colectomy might confer some protection against developing dementia in individuals with UC by eliminating chronic colonic inflammation.

This study used national Swedish register data to 1) assess the risk of developing dementia in individuals with and without UC; 2) compare this risk in those with UC who underwent colectomy with those who did not.

Methods

Study design and population

This study was based on several Swedish national registers linked through the personal identification number issued to all residents in Sweden. The Total Population Register (TPR) includes demographic information such as date of birth, sex, date of death, and migration for all individuals born since 1932 and alive in 1963 and beyond [15]. The National Patient Register (NPR) records primary and secondary diagnoses from inpatient care since 1964 and outpatient information since 2001 [16]. The Cause of Death Register (CDR) contains information on all deaths since 1952 [17]. In both the NPR and CDR, diagnoses were classified according to the International Classification of Diseases (ICD) versions 7/8/9/10. Additionally, the Prescribed Drug Register (PDR) provided data on all dispensed medication prescriptions since July 1, 2005, including prescription date and dosage, utilizing the Anatomical Therapeutic Classification (ATC) system. Lastly, the Longitudinal Integration Database for Health Insurance and Labour Market Studies register (Swedish acronym, LISA), which had been compiled annually from 1990, and the

For peer review only

Population and Housing Census (conducted every 5 years between 1960 and 1990), were used to obtain socioeconomic information.

In order to ensure that individuals included in the study had reached an age relevant for assessing dementia, we included all individuals born in Sweden who were at least 59 years between 1964 and 2018. The follow-up period to detect dementia in each individual began at age 59 years or the start of the NPR on January 1, 1964, whichever came later. We chose age 59 as the starting point because it is an age range where dementia becomes more prevalent in the population, although UC and colectomy status were also identified before this age. The follow-up extended until the occurrence of any of the following events: death, emigration, a diagnosis of dementia, or the end of the study period on December 31, 2018, whichever happened first. Individuals with less than one year of follow-up were excluded.

Patient and public involvement

No patient involved. The public was also not involved in the design, or conduct, or reporting, or dissemination plans of the research.

Exposures

In this study, we included two different exposures: the occurrence of UC, with or without colectomy. In accordance with prior research,[18] the date of diagnosis of UC was identified using ICD codes in the NPR: ICD-7: 572, ICD-8 563, 569, ICD-9 556, and ICD-10 K51. Those who had a CD diagnosis first, but followed by two or more UC diagnoses were classified as having UC from the first diagnosis. As we only had access to three-digit codes for ICD-8, differentiating between UC and CD relied on subsequent diagnoses in the same patients using ICD-9/10 codes. Individuals who were solely identified through ICD-8 codes were excluded from the

For peer review only

analysis (n = 251). Furthermore, using data until the end of follow-up, individuals were excluded if they had an initial diagnosis of UC but subsequently received at least two CD diagnoses (n=10,682) or had a CD diagnosis not followed by at least two UC diagnoses (n=19,496; Figure 1 visually depicts the process of identifying the individuals included in the analysis). Among individuals with UC, we identified individuals who underwent total colectomy and were identified using the Swedish Classification of Operation and Major Procedures codes: JFH00, JFH01, JFH10, JFH11, JFH20, JFH30, JFH33, JFH40, and JFH96. Both exposures were allowed to vary over time, that is, individuals started as nonexposed until the occurrence of each exposure separately and considered as exposed from that time onwards. Those who had UC or a colectomy before the start of follow-up were coded as exposed from the start of follow-up. We created two variables: one indicating the presence or absence of a UC diagnosis (1/0) and a second variable further breaking down people with UC into with and without colectomy and compared both groups to those without UC.

Outcome

All-cause dementia was defined as either 1) a recorded diagnoses in the NPR using ICD codes: ICD-7: 304, 305 and 306, ICD-8 290, 293.0-293.1, ICD-9 290A, 290B, 290E, 290W, 290X, 294B, 331A-C, 331X, and ICD-10 F00-F01, F02.3, F03, F05.1, F09, G30, or G31.1, 2) at least two recorded dispensed medications of any of the following anticholinesterases: N06DA01, N06DA03, N06DA04, N06DX01, N06DA02, N06DA52, or 3) a recorded entry in the Death register with the previous all-cause dementia ICD codes. Alzheimer's disease included ICD-10 codes F00, G30, ICD-9 codes 290A/B, 331A, and ICD-8 code 290, ICD-7 code 305.00. Vascular dementia included ICD-10 codes F01, ICD-9 290E, and ICD-8 codes 293.0-293.1, and ICD-7 code 306.99. Given the insidious nature of dementia, the

For peer review only

date of the onset of all-cause dementia was defined as three years before the diagnosis of dementia in the NPR or a dispensed medication and five years before the death. Individuals with a diagnosis of dementia before the start of follow-up were excluded from the analysis (n = 398; Figure 1).

Covariates

Potential confounding factors included birth year, sex, region (Norrland, Svealand, and Götaland), socioeconomic index (SEI), and the household crowding index (HCI). Using the Population and Housing Census (FOB; 1960–1990 every 10 years), we extracted information on SEI and HCI using the year closest to birth (when not available, information was taken from the census up to age 10 years). SEI was based on the occupation of the father of the household (or the mother when there was no information on the father). The HCI was calculated as the ratio of total number of individuals in the household to the number of rooms in the household.

Statistical analysis

Demographic and other individual baseline characteristics were summarized by median, proportions, and interquartile range (IQR) for individuals without UC and for those with UC that did and did not undergo colectomy. Incidence rates (IR) of dementia with 95% confidence intervals (CI) were calculated fitting a Poisson generalized linear model with the log link function and robust standard errors. A Cox proportional hazards model with age as the underlying time scale[19] was fitted to estimate hazard ratios (HRs) with 95% confidence intervals (CI) for the association between colectomy and dementia onset. Both exposures (UC and total colectomy) were modelled as time-dependent covariates, classifying individuals as exposed only after the occurrence of each exposure. Analyses were adjusted for sex, birth year, and

For peer review only

county of birth. First, we examined the association between UC (regardless of colectomy) and the risk of developing all-cause dementia. Subsequently, we separated the UC group into two subgroups based on colectomy status: those with and without a colectomy. By conducting separate analyses for each subgroup, we were able to assess associations with dementia risk for UC with and without colectomy. Shoenfeld residuals were inspected in order to assess the proportionality assumption and when violated, analyses were stratified by the variables that did not meet this assumption. Data management and statistical analyses were performed using R 4.2.2 [20].

Sensitivity analysis

Due to incomplete coverage in the NPR, the analysis was repeated including only individuals who were 60 years old after 1973 to ensure greater coverage.

Results

This cohort study comprised 4,821,488 individuals with a median (IQR) age at the start of follow-up of 59 (SD=0.71) years, of whom 2,534,500 (52.6%) were female and 2,286,988 (47.4%) were male. Among those, 48,993 (1%) had a diagnosis of UC, of whom 493 (1%) had a colectomy before or during follow-up, with a median (IQR) age at the operation of 63 (51–74) years. During a total follow-up time of 84.1 million person-years between 1964 and 2018, a diagnosis of all-cause dementia was observed in 546,387 (11.3%) individuals. A summary of the cohort demographic characteristics is presented in Table 1.

The incidence rate of all-cause dementia was 63.90 (63.73–64.07) events per 10,000 person-years in individuals without UC, 94.80 (92.04–97.64) among those

For peer review only

with UC, 95.01 (92.25–97.86) in those with UC but without colectomy, and 63.42 (40.92–98.31) for those with UC and a colectomy. Cox models adjusted for sex, birth year, and birth county showed that individuals with UC had an increased all-cause dementia risk compared with the general population (HR=1.07, 95% CI [1.04–1.10]). When we separated individuals with UC into with or without a colectomy and compared both groups to individuals without UC, dementia risk for individuals with UC but without colectomy is increased (HR=1.07 [1.04–1.10]), but the risk became nonsignificant for individuals with UC and a total colectomy (HR=0.89 [0.57–1.38]). Individuals with UC who underwent a colectomy showed a suggestive decrease in the risk of dementia (HR=0.83 [0.54–1.30]) compared to those with UC but without a colectomy, however, the wide confidence interval indicated substantial uncertainty.

Sensitivity analyses showed that after restricting analyses to those who started follow-up in 1974 and onwards, the magnitude of the association decreased compared with the main analysis but still indicated that those with UC were at increased risk for all-cause dementia (HR=1.04 [1.01–1.08]). For those with UC and without a colectomy, we observed an increased risk for all-cause dementia compared to the general cohort (HR=1.04 [1.01–1.08]) and no difference in the hazard between those with UC and a total colectomy and the general population (HR=0.92 [0.60–1.43]). Figure 2 summarizes the results of the main and sensitivity analyses.

Discussion

This large cohort study of individuals from the general population with prospectively recorded data assessed the association between UC and subsequent development of all-cause dementia and investigated the previous unexplored topic, to the best of our knowledge of the potential role of colectomy in this relationship. The

For peer review only

results indicated that individuals with UC are at slightly increased all-cause dementia risk compared with those without UC, but there was no evidence of an even greater magnitude risk in those who has a colectomy.

Recent meta-analysis evidence indicated an increased dementia risk in individuals with IBD with a pooled risk ratio of 1.35 [1.08–1.68] [14]. Our findings are consistent with this finding and mirror conclusions obtained in previous studies suggesting a relationship between UC and dementia, with a similar magnitude of the association to those obtained in Danish register studies [13] and Canadian [21] and German cohort studies [22]. Our study provides novel findings by indicating that having a total colectomy does not further increase dementia among people with UC. While our results are consistent with possible reduction of dementia risk in UC following colectomy, the interpretation remains equivocal. It is noteworthy that as colectomy signals more severe UC disease activity, as well as the inconvenience post-colectomy, there was no additional dementia risk compared to those with UC without colectomy.

While the precise aetiology of UC remains elusive, it has been proposed that a dysregulated mucosal immune response, in the context of a disturbed gut microbiota, plays a pivotal role in the onset and progression of the disease [23–26]. The intestinal immune response is a process composed of complex interactions between several cell types interacting within a specific microenvironment, the intestinal mucosa. When these interactions are perturbed by dysbiosis, such as during the onset of IBD, an excessive immune response occurs, with an aberrant mucosal immune response that involves the production of large amounts of pro-inflammatory cytokines, such as interleukins, that can lead to severe tissue damage [27] and it has been associated with several psychiatric conditions, including dementia [1–4,9–12]. A balance

For peer review only

between proinflammatory and anti-inflammatory signals is necessary for the host to avoid acute or chronic inflammation and its harmful consequences. We hypothesize that with the removal of the colon, the excessive immune response and the overproduction of pro-inflammatory cytokines cease, reducing colonic and potentially chronic systemic inflammation and, in turn, potentially reducing the risk of dementia.

Strengths and limitations

This study presents several noteworthy strengths, including a nationwide sample of over 4.8 million individuals including 84.1 million person-years over seven decades followed for an average of 17 years of observation time with prospectively recorded coverage from inpatient and outpatient services with a 79–90% positive predictive value for UC [28]. To further reduce UC misclassification, we required patients to have at least 2 consecutive UC diagnoses. There are potential limitations that should be considered. First, the estimated increased risk for dementia in people with UC may be, in part, explained by surveillance bias, where people with UC have increased contact with healthcare systems. However, those with UC and a colectomy also experience closer contact with healthcare services and we did not observe an increased risk. Second, although we obtained all-cause dementia diagnoses from inpatient and outpatient care services, misclassification is possible. In order to reduce misclassification, we retrieved all prescribed anticholinesterases used to treat dementia from the PDR from 2005 onwards (>99% coverage). However, diagnoses of dementia and UC before 2001 were based solely on inpatient data. Without outpatient data prior to 2001, there is a possibility of under-identification of dementia and UC diagnoses. It is crucial to interpret the results within the context of these data constraints and acknowledge the potential biases that may arise due to incomplete

For peer review only

data capture before the specified time periods. Nonetheless, our estimated IR (6.4 per 1,000 person-year) is smaller than those recently reported in the literature [29], highlighting potential misclassification. This misclassification –most likely non-differential– refers to individuals with dementia that are not identified leading to systematic error in the estimation of association and would bias results towards the null. Further, given the low count of individuals with UC who had a colectomy, we were not able to assess the importance of age at colectomy and whether this association varies by dementia type.

Conclusions

Our findings contribute to the growing body of literature on the increased risk of developing dementia among individuals with UC. Additionally, our study indicated that total colectomy in UC patients is not associated with an even higher magnitude increased dementia risk.

For peer review only

Author Contributions

Dr Garcia-Argibay had full access to all the data in the study and takes responsibility for the integrity of the data and the accuracy of the data analysis.

Concept and design: All authors.

Statistical analysis: Garcia-Argibay.

Acquisition, analysis, or interpretation of data: All authors.

Drafting of the manuscript: Garcia-Argibay.

Critical revision of the manuscript for important intellectual content: All authors.

Supervision: Montgomery, Hiyoshi.

Conflict of Interest Disclosures

Funding/Support

This work was supported by grant from the Swedish Research Council for Health, Working Life and Welfare (number: 2019-01236).

Role of the Funder/Sponsor

The funders had no role in the design and conduct of the study; collection, management, analysis, and interpretation of the data; preparation, review, or approval of the manuscript; and decision to submit the manuscript for publication.

Ethical approval

The study had ethical approval from the Swedish Ethical Review Authority (Dnr 2019-04755, 2020-02406, and 2022-00336-02). Requirement for informed consent was waived for the current study because it was a secondary analysis of existing data.

For peer review only

The investigation conforms to the 1964 Helsinki declaration and its later amendments or comparable ethical standards.

Data sharing

The Public Access to Information and Secrecy Act in Sweden prohibits us from making individual level data publicly available. Researchers who are interested in replicating our work can apply for individual level data at Statistics Sweden: www.scb.se/en/services/guidance-for-researchers-and-universities/.

For peer review only

References

1 Netea MG, Balkwill F, Chonchol M, *et al.* A guiding map for inflammation. *Nat Immunol*. 2017;18:826–31.

2 Bennett JM, Reeves G, Billman GE, *et al.* Inflammation–Nature's Way to Efficiently Respond to All Types of Challenges: Implications for Understanding and Managing "the Epidemic" of Chronic Diseases. *Front Med*. 2018;5:316.

3 Walker KA, Gottesman RF, Wu A, *et al.* Systemic inflammation during midlife and cognitive change over 20 years: The ARIC Study. *Neurology*. 2019;92:e1256–67.

4 Cunningham C, Hennessy E. Co-morbidity and systemic inflammation as drivers of cognitive decline: new experimental models adopting a broader paradigm in dementia research. *Alzheimers Res Ther*. 2015;7:33.

5 Pan W, P. Stone K, Hsuchou H, *et al.* Cytokine Signaling Modulates Blood-Brain Barrier Function. *Curr Pharm Des*. 2011;17:3729–40.

6 Varatharaj A, Galea I. The blood-brain barrier in systemic inflammation. *Brain Behav Immun*. 2017;60:1–12.

7 Miller AH, Haroon E, Raison CL, *et al.* CYTOKINE TARGETS IN THE BRAIN: IMPACT ON NEUROTRANSMITTERS AND NEUROCIRCUITS: Review: Cytokine Targets in the Brain. *Depress Anxiety*. 2013;30:297–306.

8 Raison CL, Capuron L, Miller AH. Cytokines sing the blues: inflammation and the pathogenesis of depression. *Trends Immunol*. 2006;27:24–31.

9 Felger JC, Lotrich FE. Inflammatory cytokines in depression: Neurobiological mechanisms and therapeutic implications. *Neuroscience*. 2013;246:199–229.

10 Miná VAL, Lacerda-Pinheiro SF, Maia LC, *et al.* The influence of inflammatory cytokines in physiopathology of suicidal behavior. *J Affect Disord*. 2015;172:219–30.

11 Tarkowski E, Blennow K, Wallin A, *et al.* Intracerebral production of tumor necrosis factor-alpha, a local neuroprotective agent, in Alzheimer disease and vascular dementia. *J Clin Immunol*. 1999;19:223–30.

12 Grimaldi LM, Casadei VM, Ferri C, *et al.* Association of early-onset Alzheimer's disease with an interleukin-1alpha gene polymorphism. *Ann Neurol*. 2000;47:361–5.

13 Rønnow Sand J, Troelsen FS, Horváth-Puhó E, *et al.* Risk of dementia in patients with inflammatory bowel disease: a Danish population-based study. *Aliment Pharmacol Ther*. 2022;56:831–43.

14 Zhang M, Shi Y, Jiang H. The risk of dementia in patients with inflammatory bowel disease: a systematic review and meta-analysis. *Int J Colorectal Dis*. 2022;37:769–75.

For peer review only

15 Ludvigsson JF, Almqvist C, Bonamy A-KE, *et al.* Registers of the Swedish total population and their use in medical research. *Eur J Epidemiol*. 2016;31:125–36. doi: 10.1007/s10654-016-0117-y

16 Ludvigsson JF, Andersson E, Ekbom A, *et al.* External review and validation of the Swedish national inpatient register. *BMC Public Health*. 2011;11:450.

17 Brooke HL, Talbäck M, Hörnblad J, *et al.* The Swedish cause of death register. *Eur J Epidemiol*. 2017;32:765–73.

18 Garcia-Argibay M, Hiyoshi A, Montgomery S. Acute appendicitis and ulcerative colitis: a population-based sibling comparison study. *BMJ Open Gastroenterol*. 2022;9:e001041.

19 Kom EL, Graubard BI, Midthune D. Time-to-Event Analysis of Longitudinal Follow-up of a Survey: Choice of the Time-scale. *Am J Epidemiol*. 1997;145:72–80.

20 R Core Team. R: A Language and Environment for Statistical Computing. 2020.

21 Bernstein CN, Nugent Z, Shaffer S, *et al.* Comorbidity before and after a diagnosis of inflammatory bowel disease. *Aliment Pharmacol Ther*. 2021;54:637–51.

22 Zingel R, Bohlken J, Kostev K. Association Between Inflammatory Bowel Disease and Dementia: A Retrospective Cohort Study. *J Alzheimers Dis JAD*. 2021;80:1471–8.

23 Targan SR, Karp LC. Defects in mucosal immunity leading to ulcerative colitis. *Immunol Rev*. 2005;206:296–305.

24 Strober W, Fuss I, Mannon P. The fundamental basis of inflammatory bowel disease. *J Clin Invest*. 2007;117:514–21.

25 Garrett WS, Lord GM, Punit S, *et al.* Communicable Ulcerative Colitis Induced by T-bet Deficiency in the Innate Immune System. *Cell*. 2007;131:33–45.

26 Chen H, Li H, Liu Z. Interplay of intestinal microbiota and mucosal immunity in inflammatory bowel disease: a relationship of frenemies. *Ther Adv Gastroenterol*. 2020;13:175628482093518.

27 Muthas D, Reznichenko A, Balendran CA, *et al.* Neutrophils in ulcerative colitis: a review of selected biomarkers and their potential therapeutic implications. *Scand J Gastroenterol*. 2017;52:125–35.

28 Jakobsson GL, Sternegård E, Olén O, *et al.* Validating inflammatory bowel disease (IBD) in the Swedish National Patient Register and the Swedish Quality Register for IBD (SWIBREG). *Scand J Gastroenterol*. 2017;52:216–21.

29 Wolters FJ, Chibnik LB, Waziry R, *et al.* Twenty-seven-year time trends in dementia incidence in Europe and the United States: The Alzheimer Cohorts Consortium. *Neurology*. 2020;95:e519–31.

For peer review only

For peer review only

Tables

Table 1. Characteristics of the cohort (N=4,821,488).

Variable	Without IBD (N=4,772,495)[1]	UC without colectomy[2] (N=48,500)[1]	UC with colectomy[2] (N=493)[1]
Median follow-up time (years)	17 (10, 25)	17 (10, 24)	13 (7, 19)
Age at the start of follow-up (years)	59 (59, 59)	59 (59, 59)	59 (59, 59)
Sex (%)			
Male	2,263,178 (47)	23,513 (48)	297 (60)
Female	2,509,317 (53)	24,987 (52)	196 (40)
Household crowding index (HCI)	1.50 (1.00, 2.00)	1.50 (1.00, 2.00)	1.50 (1.20, 2.00)
Missing	2,917,369	27,917	186
Socioeconomic index (SEI; %)			
Agriculture	289,162 (6.1)	2,991 (6.2)	43 (8.7)
High	212,356 (4.4)	2,315 (4.8)	35 (7.1)
Low	801,437 (17)	9,268 (19)	138 (28)
Medium	463,642 (9.7)	4,972 (10)	73 (15)
Unknown	2,918,081 (61)	27,919 (58)	186 (38)
Other	87,817 (1.8)	1,035 (2.1)	18 (3.7)
County (%)			
Goaland	2,126,607 (45)	22,223 (46)	190 (39)
Norrland	679,216 (14)	7,004 (14)	52 (11)
Svealand	1,657,347 (35)	17,397 (36)	232 (47)
Unknown	309,325 (6.5)	1,876 (3.9)	19 (3.9)
Median age of colectomy (years)	73 (67, 79)	0	65 (56, 72)
Without colectomy	4,771,206	48,500	0
All-cause dementia (%)	541,574 (11)	4,785 (9.9)	28 (5.7)
Alzheimer's disease	146,689 (3.1)	1261 (2.6)	7 (1.4)
Vascular dementia	70,615 (1.5)	652 (1.3)	2 (0.4)

Note. [1]Median (IQR); n (%), [2] variables defined as present from the start of follow-up.

For peer review only

Figures

Figure 1. Flowchart illustrating the screening and inclusion process of study participants based on the inclusion and exclusion criteria.

Figure 2. Forest plot of the association between UC and all-cause dementia in those with UC (with and without total colectomy) compared with unexposed individuals.

For peer review only

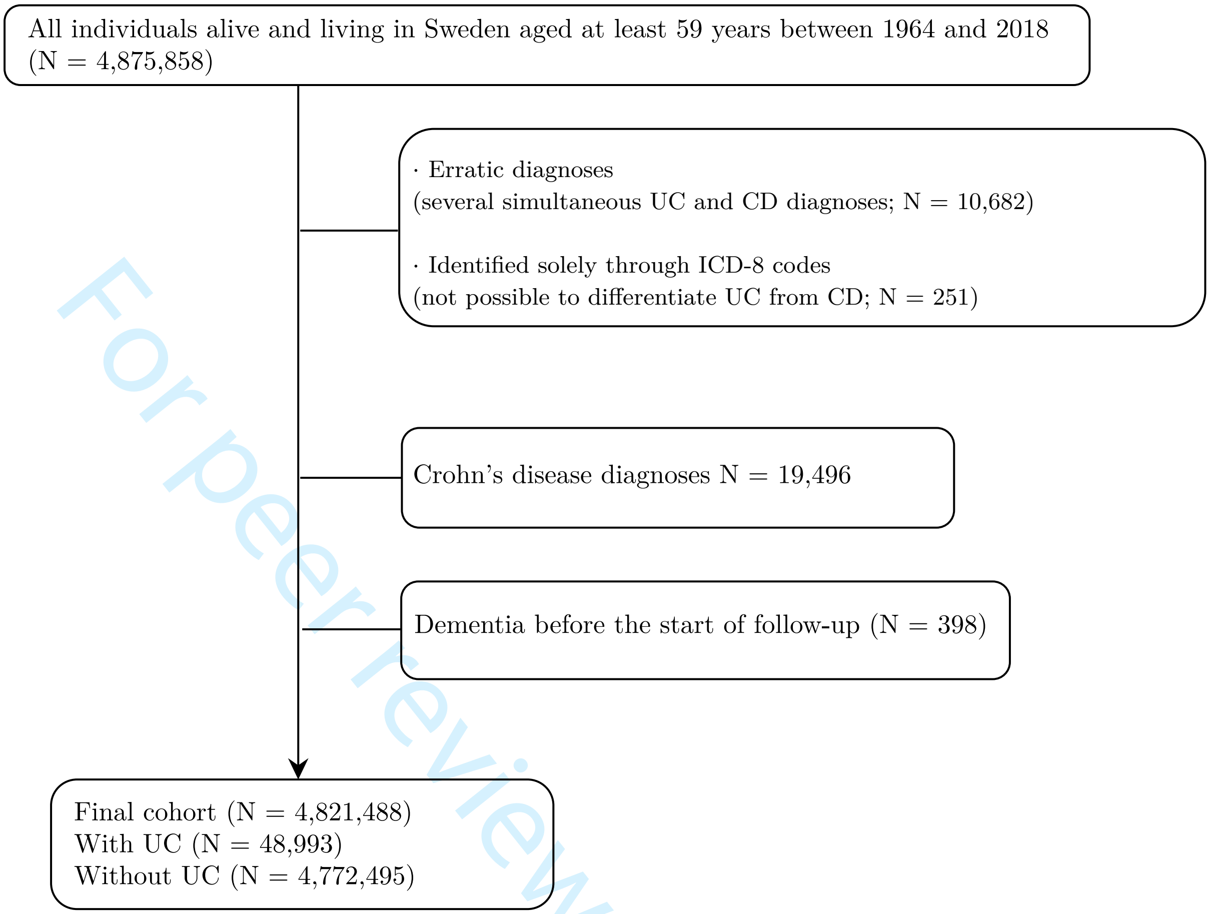

For peer review only

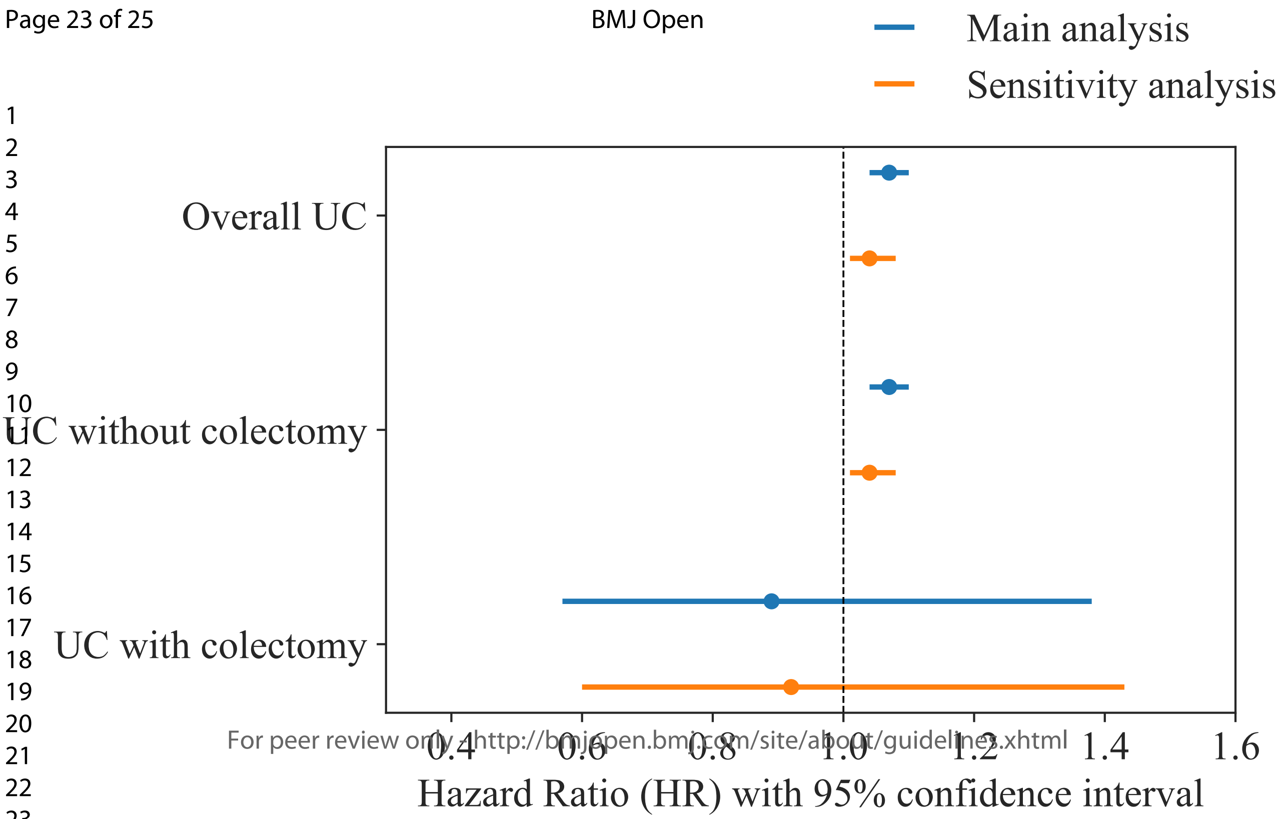
Main analysis
Sensitivity analysis
Overall UC
UC without colectomy
UC with colectomy
0.4
0.6
0.8
1.0
1.2
1.4
1.6
Hazard Ratio (HR) with 95% confidence interval

STROBE Statement—checklist of items that should be included in reports of observational studies

	Item No.	Recommendation	Page No.	Relevant text from manuscript
**Title and abstract**	1	(*a*) Indicate the study's design with a commonly used term in the title or the abstract	1	
		(*b*) Provide in the abstract an informative and balanced summary of what was done and what was found	2	
**Introduction**				
Background/rationale	2	Explain the scientific background and rationale for the investigation being reported	3-4	introduction
Objectives	3	State specific objectives, including any prespecified hypotheses	4	
**Methods**				
Study design	4	Present key elements of study design early in the paper	4	Study design and population
Setting	5	Describe the setting, locations, and relevant dates, including periods of recruitment, exposure, follow-up, and data collection	5	
Participants	6	(*a*) *Cohort study*—Give the eligibility criteria, and the sources and methods of selection of participants. Describe methods of follow-up<br>*Case-control study*—Give the eligibility criteria, and the sources and methods of case ascertainment and control selection. Give the rationale for the choice of cases and controls<br>*Cross-sectional study*—Give the eligibility criteria, and the sources and methods of selection of participants	4-5	
		(*b*) *Cohort study*—For matched studies, give matching criteria and number of exposed and unexposed<br>*Case-control study*—For matched studies, give matching criteria and the number of controls per case		
Variables	7	Clearly define all outcomes, exposures, predictors, potential confounders, and effect modifiers. Give diagnostic criteria, if applicable	4-6	Outcome, exposures, covariates
Data sources/ measurement	8*	For each variable of interest, give sources of data and details of methods of assessment (measurement). Describe comparability of assessment methods if there is more than one group	*4-6*	
Bias	9	Describe any efforts to address potential sources of bias	7	
Study size	10	Explain how the study size was arrived at	4-6	

Continued on next page

For peer review only - http://bmjopen.bmj.com/site/about/guidelines.xhtml

Quantitative variables	11	Explain how quantitative variables were handled in the analyses. If applicable, describe which groupings were chosen and why	4-6	
Statistical methods	12	(*a*) Describe all statistical methods, including those used to control for confounding	7	Statistical analysis
		(*b*) Describe any methods used to examine subgroups and interactions	7	
		(*c*) Explain how missing data were addressed	NA	
		(*d*) *Cohort study*—If applicable, explain how loss to follow-up was addressed<br>*Case-control study*—If applicable, explain how matching of cases and controls was addressed<br>*Cross-sectional study*—If applicable, describe analytical methods taking account of sampling strategy	7	
		(*e*) Describe any sensitivity analyses		
**Results**				
Participants	13*	(a) Report numbers of individuals at each stage of study—eg numbers potentially eligible, examined for eligibility, confirmed eligible, included in the study, completing follow-up, and analysed	7-8	Results
		(b) Give reasons for non-participation at each stage	7-8	
		(c) Consider use of a flow diagram	NA	
Descriptive data	14*	(a) Give characteristics of study participants (eg demographic, clinical, social) and information on exposures and potential confounders	7-8/Table1	
		(b) Indicate number of participants with missing data for each variable of interest	Table1	
		(c) *Cohort study*—Summarise follow-up time (eg, average and total amount)	7-8	
Outcome data	15*	*Cohort study*—Report numbers of outcome events or summary measures over time		
		Case-control study—Report numbers in each exposure category, or summary measures of exposure		
		Cross-sectional study—Report numbers of outcome events or summary measures		
Main results	16	(*a*) Give unadjusted estimates and, if applicable, confounder-adjusted estimates and their precision (eg, 95% confidence interval). Make clear which confounders were adjusted for and why they were included	7-8	
		(*b*) Report category boundaries when continuous variables were categorized		
		(*c*) If relevant, consider translating estimates of relative risk into absolute risk for a meaningful time period		

Continued on next page

For peer review only - http://bmjopen.bmj.com/site/about/guidelines.xhtml

Other analyses	17	Report other analyses done—eg analyses of subgroups and interactions, and sensitivity analyses	8	
**Discussion**				
Key results	18	Summarise key results with reference to study objectives	9-11	Discussion
Limitations	19	Discuss limitations of the study, taking into account sources of potential bias or imprecision. Discuss both direction and magnitude of any potential bias	10	
Interpretation	20	Give a cautious overall interpretation of results considering objectives, limitations, multiplicity of analyses, results from similar studies, and other relevant evidence	10	
Generalisability	21	Discuss the generalisability (external validity) of the study results	10	
**Other information**				
Funding	22	Give the source of funding and the role of the funders for the present study and, if applicable, for the original study on which the present article is based	12	

*Give information separately for cases and controls in case-control studies and, if applicable, for exposed and unexposed groups in cohort and cross-sectional studies.

Note: An Explanation and Elaboration article discusses each checklist item and gives methodological background and published examples of transparent reporting. The STROBE checklist is best used in conjunction with this article (freely available on the Web sites of PLoS Medicine at http://www.plosmedicine.org/, Annals of Internal Medicine at http://www.annals.org/, and Epidemiology at http://www.epidem.com/). Information on the STROBE Initiative is available at www.strobe-statement.org.

For peer review only
